# Design of α-Fe_2_O_3_ nanorods functionalized tubular NiO nanostructure for discriminating toluene molecules

**DOI:** 10.1038/srep26432

**Published:** 2016-05-19

**Authors:** Chen Wang, Tianshuang Wang, Boqun Wang, Xin Zhou, Xiaoyang Cheng, Peng Sun, Jie Zheng, Geyu Lu

**Affiliations:** 1 State Key Laboratory on Integrated Optoelectronics, College of Electronic Science and Engineering, Jilin University, Changchun 130012, People’s Republic of China

## Abstract

A novel tubular NiO nanostructure was synthesized by a facile and low-cost hydrothermal strategy and then further functionalized by decorating α-Fe_2_O_3_ nanorods. The images of electron microscopy indicated that the α-Fe_2_O_3_ nanorods were assembled epitaxially on the surfaces of NiO nanotubes to form α-Fe_2_O_3_/NiO nanotubes. As a proof-of-concept demonstration of the function, gas sensing devices were fabricated from as-prepared α-Fe_2_O_3_/NiO nanotubes, and showed enhanced gas response and excellent selectivity toward toluene, giving a response of 8.8 to 5 ppm target gas, which was about 7.8 times higher than that of pure NiO nanotubes at 275 °C. The improved gas sensing performance of α-Fe_2_O_3_/NiO nanotubes could be attributed to the unique tubular morphology features, p-n heterojunctions and the synergetic behavior of α-Fe_2_O_3_ and NiO.

With the development of social economy and the changes of people’s living style, the activities of producing, working, learning and entertainment are more transformed from the outdoors to the indoors. Therefore, indoor air quality is significantly important for people’s health. However, the decorative materials can release some harmful and toxic volatile organic compounds (VOCs), including benzene, toluene, formaldehyde and so on. Among them, toluene as colorless and volatile gas molecules are stimulus to the skin and mucous membranes, which could cause great harm to human body upon long-term exposure. For this reason, selectively and efficiently discriminating traces of toluene gas molecules has important realistic meaning.

At present, resistive type gas sensors^1^,^2^,^3^,^4^,^5^,^6^,^7^,^8^,^9^ using various metal oxide semiconductors including SnO_2_^1^, In_2_O_3_^2^, WO_3_^3^, TiO_2_^4^, CuO^5^ and Co_3_O_4_^6^ as gas sensing materials have received extensive attentions due to their superior stability, low cost and simple preparation. Although promising results of the gas sensing performance of semiconductor oxides have been reported, the design and development of highly sensitive and selective sensing materials remains an on-going challenge for gas sensors. Many studies have been conducted in order to improve the gas sensing properties of metal oxides by growth of novel morphologies and structures with controllable dimension since the fact that the sensing properties of oxides are affected by their morphology. However, doing that only by adjusting the morphologies and structures of sensing materials is far from enough. Therefore, during the past decade, tremendous efforts apart from the control of morphology have been devoted to modifying semiconductor oxides sensing materials, including (i) noble metal loading^10^,^11^; (ii) transition metals doping^12^,^13^; (iii) the formation of n-type/p-type heterojunctions^14^. Recently, the construction of hetero-structures by combining with different semiconductor oxides has been found to result in significantly enhanced gas response during sensing. In this regard, design and preparation of composites with unique heterostructures will be increasingly important in the development of high performance gas sensors.

Nickel oxide (NiO) and ferric oxide (α-Fe_2_O_3_), both important versatile materials, have attracted enormous interests because of their potential applications in dye-sensitized solar cells^15^,^16^, lithium ion batteries^17^,^18^, electrochemical capacitors^19^,^20^, catalysts^21^,^22^, gas sensors^23^,^24^, etc. It has been demonstrated that the heterojunction nanostructures by combining NiO and α-Fe_2_O_3_ can greatly enhance their application performance^25^,^26^. Therefore, α-Fe_2_O_3_/NiO heterostructure with various architectures have been prepared through different strategies. While the solution approaches have proven to be among the most effective route to finely tailor semiconductor composites with varying compositional and architectural complexity^25^,^27^. In our previous research^25^, for the first time, we reported the feasible strategy of fabricating hierarchical flower-like α-Fe_2_O_3_/NiO microspheres to improve the gas sensing performance. However, a number of α-Fe_2_O_3_ nanorods prefer existing as urchin-like polycrystals to absolutely compositing with flower-like NiO microspheres probably due to the overly complicated morphology of NiO. The sensor based on α-Fe_2_O_3_/NiO heterostructure performed the sensing behavior of p-type semiconductor sensor which the sensor resistance increased when the reducing gas existed, while the isolated α-Fe_2_O_3_ polycrystals as an n-type semiconductor would lead to the decrease of the sensor resistance on exposure to reducing gas. Meanwhile, the formation of the isolated α-Fe_2_O_3_ polycrystals means the decrease of the amount of α-Fe_2_O_3_/NiO heterojunctions. These could cause adverse impacts on gas sensing performance. Accordingly, it is necessary to deeply investigate the fabrication of α-Fe_2_O_3_/NiO heterostructure aiming to inhibit the formation of the isolated α-Fe_2_O_3_ polycrystals in order to further enhance the gas sensing performance of α-Fe_2_O_3_/NiO heterostructure.

In this work, we developed a facile solution method to prepare NiO nanotube structure composed of numerous primary nanoparticles. Subsequently, α-Fe_2_O_3_ nanorods as the branches directly and epitaxially grew on NiO nanotube surface to form trepang-like α-Fe_2_O_3_/NiO heterostructure eventually. Interestingly, the as-synthesized α-Fe_2_O_3_/NiO heterostructure exhibited excellent selectivity and high response to toluene in contrast to the primary NiO nanotubes, which demonstrated their potential value as the sensing material of a superior gas sensor.

## Results

### Structural characterization

**Figure 1:**
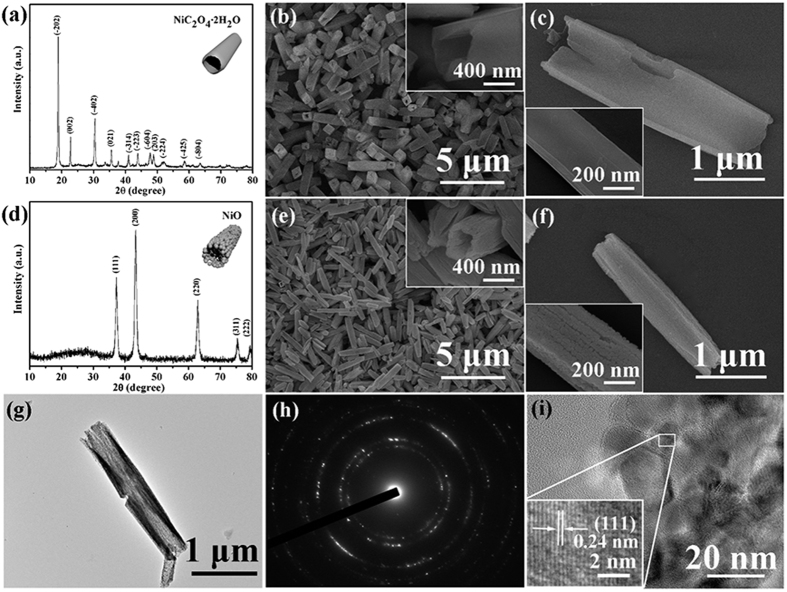
(**a**) XRD pattern of NiC_2_O_4_·2H_2_O. (**b**,**c**) Typical FESEM images of NiC_2_O_4_·2H_2_O nanotubes. (**d**) XRD pattern of NiO. (**e,f**) Typical FESEM images of NiO nanotubes. (**g**) The TEM image of a single NiO nanotube. (**h**) Corresponding SAED pattern of NiO nanotubes. (**i**) HRTEM image of NiO nanotubes.

The formation of the α-Fe_2_O_3_/NiO nanotubes was based on two-step hydrothermal processes. First, we synthesized NiC_2_O_4_·2H_2_O nanotubes via a facile hydrothermal method. The X-ray diffraction pattern (Fig. 1a) of obtained nanotubes reveals that all the diffraction peaks were consistent with the monoclinic NiC_2_O_4_·2H_2_O (JCPDS Card No. 25-0581). The morphological characteristics of the as-synthesized NiC_2_O_4_·2H_2_O were observed by FESEM. As shown in Fig. 1b, a low-magnification FESEM image, the NiC_2_O_4_·2H_2_O nanotubes were produced on a large scale with good dispersity and uniform size. Meanwhile, no other morphologies were observed. The inset of Fig. 1b confirmed that the end face was quasi-quadrilateral. Figure 1c presents a single NiC_2_O_4_·2H_2_O nanotube, which had a typical length of ~3.3 μm and a diameter of ~0.9 μm. Besides, a high-magnification FESEM image in the inset of Fig. 1c shows the highly smooth surfaces over the whole NiC_2_O_4_·2H_2_O nanotubes.

After annealed in air, the NiC_2_O_4_·2H_2_O nanotubes were turned into NiO nanotubes. The XRD pattern of the NiO nanotubes is shown in Fig. 1d, which was matched well with the face-centered cubic phase of NiO (JCPDS Card No. 47-1049). The panoramic FESEM image of the NiO nanotubes (Fig. 1e and the inset) demonstrates that the tubular morphology was maintained. However, the sizes of the NiO nanotubes were decreased compared with the NiC_2_O_4_·2H_2_O nanotubes, which could be verified by a typical individual NiO nanotube with a length of ~2.4 μm and a diameter of ~0.5 μm, as shown in Fig. 1f. Moreover, the wall of the NiO nanotube was constructed from many small nanoparticles (the inset of Fig. 1f) instead of the smooth surface before. Figure 1g shows a typical transmission electron microscopy (TEM) image of a single NiO nanotube, which indicates that the wall of the NiO tube was getting thinner from the middle to both ends. The corresponding selected area electron diffraction (SAED) pattern in Fig. 1h revealed the polycrystalline feature of the NiO nanotubes. The high-resolution TEM (HRTEM) image (Fig. 1i), which taken near the edge of the nanotube, shows that the sizes of nanoparticles were less than 20 nm and displays distinct lattice fringe with the lattice spacing of 0.24 nm, corresponding to the (111) lattice plane of the cubic NiO.

**Figure 2:**
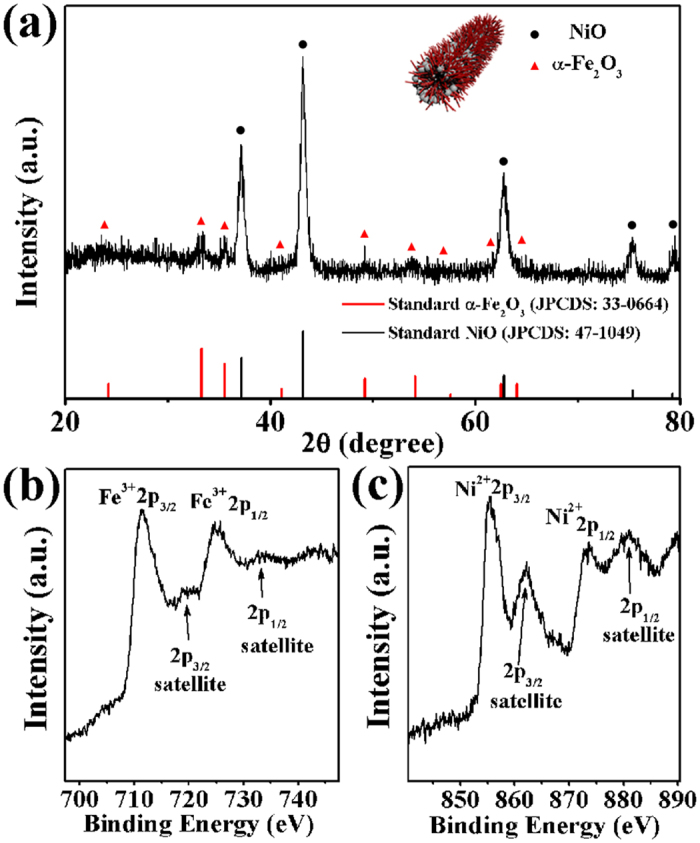
(**a**) XRD pattern of α-Fe_2_O_3_/NiO nanotubes. (**b**,**c**) XPS patterns of the Fe 2p and Ni 2p regions of the α-Fe_2_O_3_/NiO nanotubes, respectively.

Using above NiO nanotubes as the stem structures, we adopted the second hydrothermal method to grow α-Fe_2_O_3_ nanorods on the surfaces of NiO nanotubes. The crystallographic structure and phase purity of as-prepared α-Fe_2_O_3_/NiO nanotubes were examined by powder X-ray diffraction (XRD) as shown in Fig. 2a. All the diffraction peaks matched well with the standard XRD patterns of the face-centered cubic phase of NiO (JCPDS Card No. 47-1049) and the rhombohedral α-Fe_2_O_3_ (JCPDS Card No. 33-0664). No additional peaks were found in the XRD pattern, which indicates the high purity of α-Fe_2_O_3_/NiO nanotubes.

To further characterize the composition of the final α-Fe_2_O_3_/NiO nanotubes, X-ray photoelectron spectroscopy (XPS) was employed. It can be seen from the XPS survey spectrum (see Supplementary Information-Fig. S1) that the product contained Ni, Fe, O and C elements. The C signal might be attributed to adventitious hydrocarbon. Detailed scans of the Fe-2p range and the Ni-2p range are illustrated in Fig. 2b,c, respectively. As shown in Fig. 2b, Fe-2p_2/3_, Fe-2p_1/2_, and their satellites peaks were centered at 711.4, 719.6, 725.7, and 734.1 eV, respectively, which were in conformity with the electronic state of α-Fe_2_O_3_^28^. For the Ni-2p spectra (Fig. 2c), the Ni-2p signal could be also divided into four peaks. The binding energy at 855.3 eV was assigned to the Ni-2p_3/2_ peak, and its satellite peak appeared at 862.1 eV. The 873.7 and 880.1 eV peaks were attributed to Ni-2p_1/2_, and its satellite. The Ni-2p_3/2_ peaks were assigned to Ni^2+ ^29. The results further indicated that the final product was composed of α-Fe_2_O_3_ and NiO.

**Figure 3:**
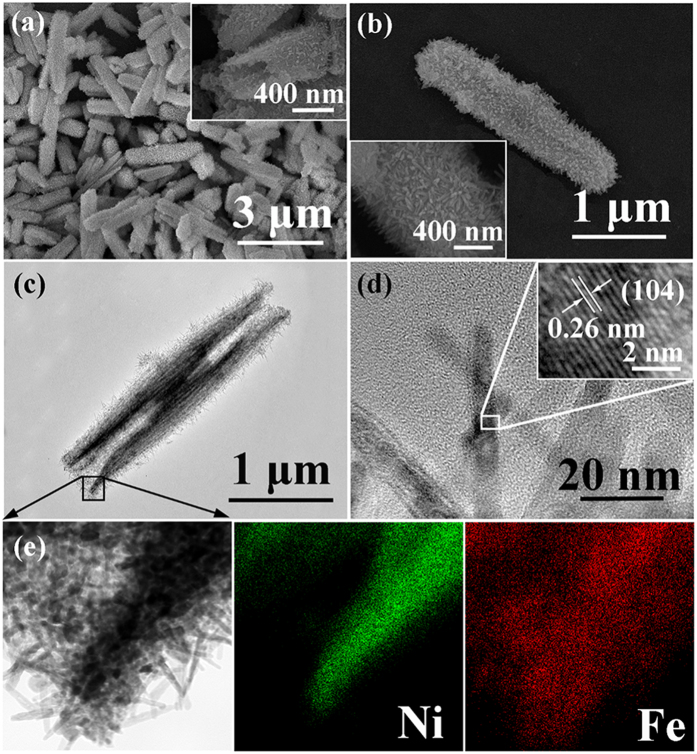
(**a**,**b**) Typical FESEM images of the α-Fe_2_O_3_/NiO nanotubes. (**c**) Typical TEM image of a single α-Fe_2_O_3_/NiO nanotube. (**d**) HRTEM image of the α-Fe_2_O_3_ nanorods. (**e**) The energy dispersive X-ray spectroscopic (EDS) elemental mapping images of an end of the α-Fe_2_O_3_/NiO nanotube from panel c.

The morphology of the α-Fe_2_O_3_/NiO nanotubes can be observed in Figure 3. Figure 3a,b show low- and high-magnification SEM images of the α-Fe_2_O_3_/NiO nanotubes, from which the tubular architectures were maintained after the second hydrothermal process and no isolated α-Fe_2_O_3_ polycrystals could be observed. From Fig. 3b, it can be observed that the single α-Fe_2_O_3_/NiO nanotube with a typical length of ~2.5 μm and a diameter of ~0.6 μm, was a little bigger than the pure NiO nanotubes due to the α-Fe_2_O_3_ nanorods branches, and the α-Fe_2_O_3_ nanorods as the branches grew radially out from the surfaces of NiO nanotubes to form the heterostructure. When observed under a transmission electron microscope (TEM), a distinct tubular structure could be clearly identified (Fig. 3c). Further crystal structural features of the α-Fe_2_O_3_ nanorods were characterized by using HRTEM. As shown in Fig. 3d, the α-Fe_2_O_3_ nanorods were single-crystalline and the measured lattice fringe with interplanar spacing of 0.26 nm well corresponded to the (104) plane of the rhombohedral α-Fe_2_O_3_. The energy dispersive X-ray spectroscopic (EDS) elemental mapping (Fig. 3e) of an end of the α-Fe_2_O_3_/NiO nanotube from panel c clearly confirms the composition of the product. Obviously, the stem was NiO material and the branch was α-Fe_2_O_3_ according to the spatial distribution of Ni and Fe signals, which were consistent with the EDS spectrum (see Supplementary Information-Fig. S2).

### Gas sensing properties

In order to better illustrate the promotion of α-Fe_2_O_3_/NiO heterostructure to gas sensing characteristics, the sensing mechanism is firstly introduced here. The gas sensing mechanism of the metal oxides gas sensors is that the interactions between the surface adsorbed oxygen species and target gases lead to a change in the electrical conductivity^30^. It is well known that, NiO as a nonstoichiometric oxide is semiconductor because of inherent defects. In order to verify the conduction type of as-prepared NiO, here, we adopted thermal probe method^31^ to measure the thermoelectromotive force of NiO under 200 °C to 450 °C (the detail measurement procedure and results can be seen in Supplementary Information-S1 and Fig. S3). Obviously, the measured thermoelectromotive force was negative, which evidenced that NiO is p-type semiconductor with holes as the main species of charge carriers. In general, at an enhanced temperature ranging from 100–500 °C (the typical operating temperature range for metal oxides gas sensor), ambient oxygen in the form of oxygen ions (O_2_^−^, O^−^ and O^2−^) are chemisorbed on the surface of NiO by capturing electrons from NiO and leads to the formation of an hole accumulation layer near the surface of NiO. Such result will increase the concentration of holes in case of NiO. When toluene gas is present, the toluene molecules can be oxidized by reacting with chemisorbed ionized oxygen anions and injects electrons into NiO to recombine with holes, which determines a decrease of the concentration of holes. As a consequence, the sensor resistance increases in comparison with the situation of pure air.

**Figure 4:**
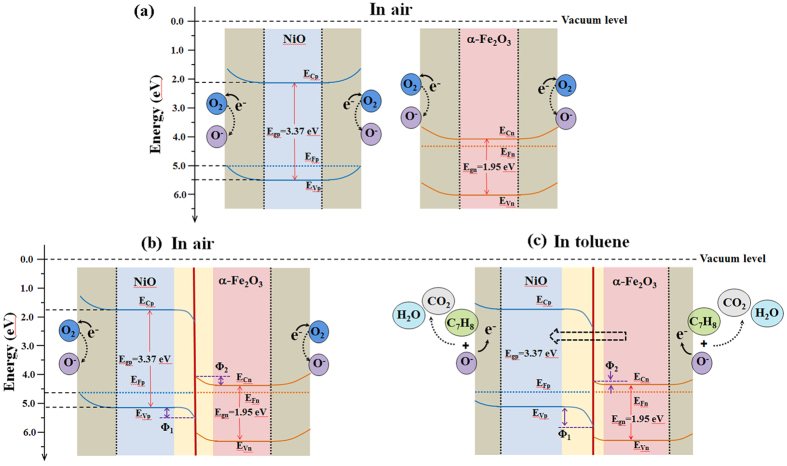
Energy band structures. (**a**) NiO and α-Fe_2_O_3_ in air before combination; (**b**) α-Fe_2_O_3_/NiO heterojunction in air; (**c**) -Fe_2_O_3_/NiO heterojunction in toluene.

The significant enhancement of the gas response to toluene for the sensor based on α-Fe_2_O_3_/NiO nanotubes should be understood in relation to the p-n heterojunction between α-Fe_2_O_3_ and NiO. It has been confirmed that the gas response of the NiO-based gas sensor can be improved significantly via adjusting the background hole concentration to lower using the doping method^12^,^30^,^32^,^33^. Similarly, in this study, the enhanced gas response of the α-Fe_2_O_3_/NiO nanotubes can also be partly explained by the variation in hole concentration. The distribution of n-type α-Fe_2_O_3_ as the branches grown epitaxially on the p-type NiO indicates that a large number of p-n junctions will be formed. The energy band structure of α-Fe_2_O_3_/NiO heterojunction can be seen in Fig. 4. First, we used (α)^2^(hʋ)^2^~hʋ relation curve to calculate the energy band gap of NiO and α-Fe_2_O_3_ by UV-vis spectra^34^,^35^, where α is the absorption coefficient, hʋ is the photon energy. The extrapolation of the straight line to the abscissa gives the band gap (E_g_). As shown in Supplementary Information-Fig. S4, the values of E_g_ were 3.37 eV and 1.95 eV for NiO and α-Fe_2_O_3_, respectively. Then we referred some references^36^,^37^,^38^,^39^ to get the Fermi level (NiO: 5.0 eV, α-Fe_2_O_3_:4.39 eV), valence band of NiO (5.5 eV), and conduction band of α-Fe_2_O_3_ (4.09 eV). According to above data, the energy band structures of NiO and α-Fe_2_O_3_ in air before combination can be drawn as Fig. 4a. After they constitute heterojunction, the electrons will transfer from α-Fe_2_O_3_ to NiO while the holes transfer in the opposite direction. This will cause the amount of holes in NiO to decrease. The space charge region is formed in the region nearby the p-n interfaces, as shown in Fig. 4b. So an electric field will be generated in the space charge region. At this point, the electric potential changes in the space charge region. As a consequence, the energy bands in the side of NiO bend downwards. The energy bands in the side of α-Fe_2_O_3_ bend upwards. The variation values of energy bands are Φ_1_ and Φ_2_ in the sides of NiO and α-Fe_2_O_3_, respectively. Meanwhile, a large number of interface states with the electron donor function will be formed due to the lattice mismatch existed in the interface of the heterojunction because of the different lattice constants of two materials, which will further decrease the number of holes in NiO. This means that the hole concentration can be adjusted by decorating α-Fe_2_O_3_ nanorods on the surface of NiO nanotubes and the abilities of absorbing and ionizing oxygen species are also improved, which indicate that toluene molecules can be oxidized to cause the significant change of the electrical resistance of the gas sensor. Moreover, the reaction between the oxygen species and toluene molecules will occur on the surface of α-Fe_2_O_3_ nanorods, leading to the release of electrons trapped in the ionized oxygen species back into the α-Fe_2_O_3_. At the moment, because potential barrier Φ_2_ located in the side of α-Fe_2_O_3_ become lower, extra electrons will transfer from α-Fe_2_O_3_ to NiO, thereby widening the barrier width and heightening the potential barrier Φ_1_, as shown in Fig. 4c. Consequently, the sensor resistance get much larger, which further promote the gas response. In addition, the larger surface area of α-Fe_2_O_3_/NiO nanotubes (44.3 m^2^ g^−1^) compared with pure NiO nanotubes (41.7 m^2^ g^−1^) (see Supplementary Information-Fig. S5) could provide more active sites, and thus sensing materials can chemisorbed more oxygen. This will further improve the gas response of the sensor based on α-Fe_2_O_3_/NiO nanotubes.

**Figure 5:**
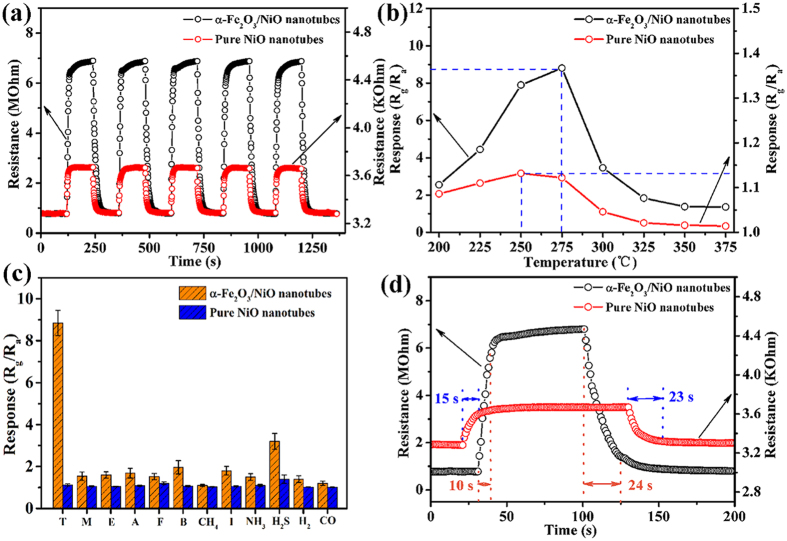
(**a**) Response curves of the α-Fe_2_O_3_/NiO nanotubes and pure NiO nanotubes to 5 ppm of toluene at 275 °C. (**b**) Responses of the α-Fe_2_O_3_/NiO nanotubes and pure NiO nanotubes vs operating temperature to 5 ppm of toluene. (**c**) Selectivities of the α-Fe_2_O_3_/NiO nanotubes and pure NiO nanotubes to 5 ppm of various gases (T, toluene; M, methanol; E, ethanol; A, acetone; F, formaldehyde; B, benzene; I, isopropanol) at 275 °C. (**d**) Responses transients of the α-Fe_2_O_3_/NiO nanotubes and pure NiO nanotubes to 5 ppm of toluene at 275 °C.

Afterwards, we compared the gas sensing properties between α-Fe_2_O_3_/NiO nanotubes and pure NiO nanotubes. The dynamic response curves of the gas sensors to 5 ppm of toluene at 275 °C are shown in Fig. 5a. For two gas sensors, the resistances increased abruptly upon exposure to toluene, which were consistent with the gas sensing characteristics of p-type oxide semiconductors. After toluene was removed, the resistances decreased and nearly recovered their initial values. The response of the sensor based on α-Fe_2_O_3_/NiO nanotubes to 5 ppm of toluene was 8.8 at 275 °C, while the response of the sensor based on pure NiO nanotubes was only 1.1. Obviously, the gas response was enhanced by fabricating α-Fe_2_O_3_/NiO heterostructure and two gas sensors showed good reversibility following multiple cycles. Besides, it can be seen that the enhanced gas response was accompanied by the obvious increase in the initial resistance of the sensor. The initial resistance of the sensor based on α-Fe_2_O_3_/NiO nanotubes was 0.78 MΩ, which was ~238 times higher than that of pure NiO nanotubes (3.28 kΩ) in air at 275 °C. This result might be connected with the space charge region of the p-n heterojunction between p-type NiO and n-type α-Fe_2_O_3_.

It is well known that the gas response was strongly dependent on the operating temperature for semiconductor oxide gas sensor. Figure 5b shows the responses of two gas sensors to 5 ppm of toluene at different operating temperatures ranging from 200 to 375 °C. We could observe volcano-shaped correlation between gas response and operating temperature. The response of the sensor based on α-Fe_2_O_3_/NiO nanotubes to 5 ppm of toluene increased from 2.55 to 8.81 with increasing the operating temperature from 200 °C to 275 °C. After that, the response decreased to 1.36 with further raising the operating temperature to 375 °C. Clearly, the optimal operating temperature of the sensor based on α-Fe_2_O_3_/NiO nanotubes was 275 °C. The similar relationship could be observed in the case of the sensor based on pure NiO nanotubes, which the maximum was 1.13 at the different optimal operating temperature of 250 °C. The difference of the optimal operating temperature among two kinds of gas sensors could be ascribed to the differences in the catalytic functions of α-Fe_2_O_3_ and NiO at different temperatures.

Selectivity is another important parameter for practical applications of the gas sensor. We investigated the responses of two gas sensors to 5 ppm of toluene, methanol, ethanol, acetone, formaldehyde, benzene, methane, isopropanol, NH_3_, H_2_S, H_2_ and CO at 275 °C (Fig. 5c), which indicates that the responses of the sensor based on α-Fe_2_O_3_/NiO nanotubes were all improved compared with pure NiO nanotubes to all target gases we tested. In addition, the sensor based on α-Fe_2_O_3_/NiO nanotubes had the highest response to toluene among all tested gases, and the response was 3.38–5.80 times higher than other tested gases. While the responses of the sensor based on pure NiO nanotubes were only 1.02–1.40 to all tested gases. The results present the excellent selectivity to toluene of the sensor based on α-Fe_2_O_3_/NiO nanotubes, which can satisfy the practical application for discriminating toluene gas molecules especially under low concentration.

In real-time monitoring, rapid response and recovery to toxic and hazardous gases were most important factors of gas sensor. Figure 5d depicts the response transients of the sensors based on α-Fe_2_O_3_/NiO nanotubes and pure NiO nanotubes exposed to 5 ppm of toluene at 275 °C. The response time of the sensors based on α-Fe_2_O_3_/NiO nanotubes was 10 s, which was shorter than that of pure NiO nanotubes (15 s). The recovery times of two sensors were almost equal. The rapid and effective gas diffusion might be obtained because porous and permeable tubular structures of both two materials. In addition, it is well known that the essence of metal oxide semiconductor gas sensor is the catalytic reaction between target gas molecules and chemisorbed oxygen occurred on the surface of sensing materials. According to the previous researches^21^,^40^,^41^,^42^, NiO and α-Fe_2_O_3_ both have excellent catalytic activity for oxidizing toluene. Therefore, after incorporating NiO and α-Fe_2_O_3_ into a heterostructure, the oxidation of toluene could be obviously facilitated, which might be ascribed to the increase of chemisorbed oxygen ions and surface active sites. That is, the synergetic catalysis actions of NiO and α-Fe_2_O_3_ for the oxidation of toluene were the important factors for promoting gas response speed.

**Figure 6:**
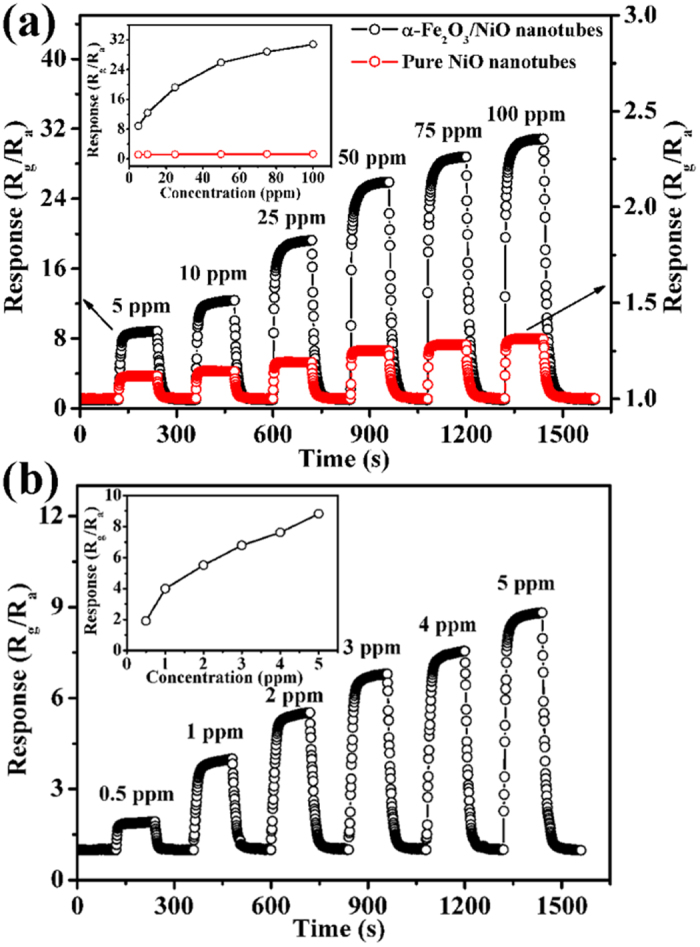
Dynamical response-recovery curves of (**a**) The α-Fe_2_O_3_/NiO nanotubes and pure NiO nanotubes to different concentrations of toluene from 5 to 100 ppm (**b**) The α-Fe_2_O_3_/NiO nanotubes to low concentrations of toluene from 0.5 to 5 ppm at 275 °C.

Figure 6a shows the dynamic response-recovery curves of two sensors to different concentrations of toluene from 5 to 100 ppm at 275 °C. It can be seen that the corresponding responses increased with increasing the concentration of toluene. The gas responses of the sensor based on α-Fe_2_O_3_/NiO nanotubes to 5, 10, 25, 50, 75 and 100 ppm of toluene were 8.8, 12.4, 19.2, 25.9, 28.8 and 30.8 respectively, which were much higher than those of pure NiO nanotubes (the response values were from 1.01 to 1.3 to the concentration of toluene from 5 to 100 ppm). The line graph of the response as the function of the concentration of toluene at 275 °C is shown in the inset of Fig. 6a. It is clearly that the response amplitude of the sensor based on α-Fe_2_O_3_/NiO nanotubes was highly dependent on toluene concentration, while there was not remarkable increase in the response of the sensor based on pure NiO nanotubes. Moreover, we further investigated the gas sensing performance of the sensor based on α-Fe_2_O_3_/NiO nanotubes to lower concentrations of toluene at 275 °C. From Fig. 6b, the gas responses of the sensor to 0.5, 1, 2, 3 and 4 ppm of toluene were 1.9, 4.0, 5.5, 6.8 and 7.6, respectively. This result obviously shows that the sensor based on α-Fe_2_O_3_/NiO nanotubes possessed very low detection limit. Simultaneously, the line graph in the inset of Fig. 6b indicated the sensor also had excellent linear responses to low concentrations of toluene. These results confirm that the sensor based on α-Fe_2_O_3_/NiO heterostructure nanotubes possessed better sensing performance to toluene than that of hierarchical flower-like α-Fe_2_O_3_/NiO microspheres reported in our previous work^25^. This phenomenon can be explained by the degree of completeness for the assembly between α-Fe_2_O_3_ and NiO because of the excellently assembled characteristic of 1-dimension nanomaterial as stem structure, which demonstrates our previous hypothesis about the adverse impacts of the isolated α-Fe_2_O_3_ polycrystals on gas sensing performance and thus further enhance the gas response to toluene.

## Discussion

In summary, unique α-Fe_2_O_3_ functionalized NiO heterostructure nanotubes constructed with α-Fe_2_O_3_ nanorods as the branch and NiO nanotubes as the stem were successfully synthesized using the facile and controllable hydrothermal approaches. As a proof-of-concept demonstration of the function, such α-Fe_2_O_3_/NiO nanotubes were used as the sensing material of gas sensor. In comparison to single component (NiO nanotubes), the heterostructure composite has demonstrated superior sensing performance to toluene. The unique tubular morphology features, p-n heterojunctions and the synergetic actions of α-Fe_2_O_3_ and NiO could be responsible for the improvement of sensing properties.

## Methods

### Preparation of NiO nanotubes

NiO nanotubes were prepared by a hydrothermal method. The procedure was briefly described as follows. 0.4754 g of NiCl_2_·6H_2_O and 0.0630 g of oxalic acid were dissolved in a mixed solution containing 9 mL of deionized water and 16 mL of glycerol to form a clear solution. The solution was then transferred into a 40 mL Teflon-lined stainless-steel autoclave and kept at 150 °C for 12 h. After the autoclave was cooled down to room temperature, the precipitate was centrifuged and washed with deionized water and ethanol several times, and then dried at 80 °C for 24 h. Finally, the NiO nanotubes were obtained by annealing above precipitate at 400 °C for 1 h in air.

### Preparation of α-Fe_2_O_3_/NiO nanotubes

The α-Fe_2_O_3_/NiO nanotubes were synthesized via the second step hydrothermal process. First, 10 mg of the obtained NiO nanotubes were dispersed in 16 mL of deionized water with ultrasonic for 5 min. Then 8.6 mg of FeCl_3_·6H_2_O and 10.3 mg of Na_2_SO_4_·10H_2_O were added into above suspension in turn. After that the mixture was poured into a 40 mL Teflon-lined stainless-steel autoclave and heated at 120 °C for 2.5 h. The resulting products were collected and washed with deionized water and ethanol by centrifugation several times, and then dried at 80 °C in air for 24 h. The α-Fe_2_O_3_/NiO nanotubes were obtained eventually by calcining at 450 °C for 2 h in a muffle furnace.

### Characterization

XRD patterns were recorded on a Rigaku TTRIII X-ray diffractometer operated at 40 kV and 200 mA with Cu Kα radiation with a wavelength of 1.5406 Å. The field emission scanning electron microscopy (FESEM) images were obtained on a JSM-7500F (JEOL) microscope operating at 15 kV. Transmission electron microscopy (TEM) and high-resolution TEM images were recorded on a JEM-2200FS (JEOL) operating at 200 kV. The energy dispersive X-ray spectroscopic (EDS) elemental mapping and spectrum were investigated by the TEM attachment. The specific surface area was estimated from the Brunauer-Emmett-Teller (BET) measurements by using a Micromeritics Gemini VII apparatus (Surface Area and Porosity System).

### Fabrication and measurement of gas sensor

The detail of the sensor fabrication and the testing process were described as follows^43^. The as-prepared NiO or α-Fe_2_O_3_/NiO nanotubes were dispersed in deionized water to form paste and then coated them on the ceramic tube (length: 4 mm, external diameter: 1.2 mm, and internal diameter: 0.8 mm) with a pair of Au electrodes, which each electrode was connected with two Pt wires. Then the coated device was sintered at 400 °C for 2 h after the paste was become dry. After that a Ni-Cr alloy coil heater was inserted to the alumina tube. The operating temperature was controlled by adjusting the heating current flowed through the heater. The schematic of the fabricated gas sensor can be shown in Supplementary Information-Fig. S6. In the testing procedure of gas sensor, a certain amount of test gases were injected into the airtight chamber. The carrier gas is high purity air (21 vol.% O_2_ and 79 vol.% N_2_). The sensor resistances in different environmental atmospheres were measured. The gas response of the sensor (p-type, reducing gas) was defined as R_g_/R_a_, which R_a_ and R_g_ were the electrical resistance of the gas sensor exposed to pure air and in the presence of target gases, respectively. The response and recovery times were defined as the time taken by the resistance value changes to 90% in the case of adsorption and desorption, respectively.

## Additional Information

**How to cite this article**: Wang, C. *et al*. Design of α-Fe_2_O_3_ nanorods functionalized tubular NiO nanostructure for discriminating toluene molecules. *Sci. Rep.* **6**, 26432; doi: 10.1038/srep26432 (2016).

## Supplementary Material

Supplementary Information
