# Supplementary material for: Design of α-Fe2O3 nanorods functionalized tubular NiO nanostructure for discriminating toluene molecules
Source: Sci Rep. 2016 May 19;6:26432. doi: 10.1038/srep26432 (PMC4872228; doi:10.1038/srep26432)
Supplement: Supplementary Information [file srep26432-s1.doc]

**Supplementary Information**

**Design of α-Fe_2_O_3_ nanorods functionalized tubular NiO nanostructure for discriminating toluene molecules**

**Chen Wang, Tianshuang Wang, Boqun Wang, Xin Zhou, Xiaoyang Cheng, Peng Sun**^*^**, Jie Zheng, Geyu Lu**^*^

State Key Laboratory on Integrated Optoelectronics, College of Electronic Science and Engineering, Jilin University, Changchun 130012, People’s Republic of China.

*Correspondence and requests for materials should be addressed to P.S. (Email: spmaster2008@163.com) or G.L. (Email: lugy@jlu.edu.cn).

**Contents:**

**Fig. S1.** XPS survey spectrum of the α-Fe_2_O_3_/NiO nanotubes.

**Fig. S2.** EDS spectrum of the α-Fe_2_O_3_/NiO nanotubes.

**Fig. S3.** (a) Schematic diagram of the thermal probe method; (b) Measured thermoelectromotive force with the temperature of the hot side ranging from 200 °C to 450 °C.

**Fig. S4.** Relationship between (α)^2^(hʋ)^2^ and hʋ for NiO (a) and α-Fe_2_O_3_ (b).

**Fig. S5.** N_2_ adsorption-desorption isotherms of (a) pure NiO and (b) α-Fe_2_O_3_/NiO nanotubes.

**Fig. S6.** Schematic diagram of the gas sensor.

**S1.** Measurement of the conduction type of as-prepared NiO by thermal probe method.


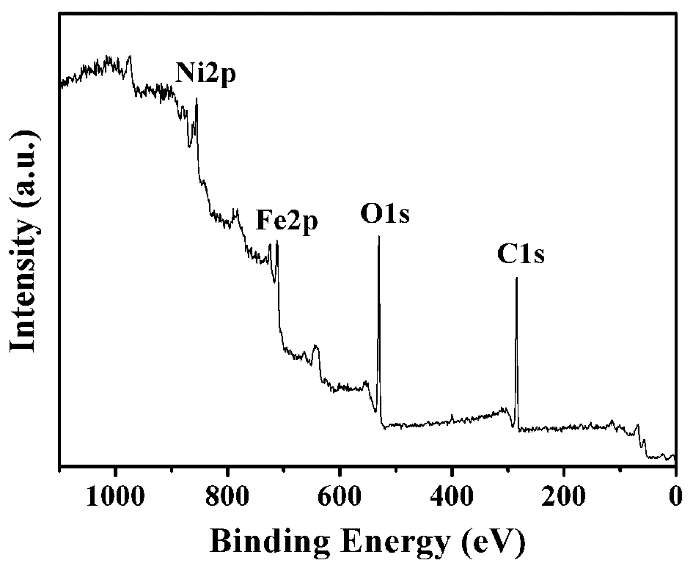


**Fig. S1.** XPS survey spectrum of the α-Fe_2_O_3_/NiO nanotubes.


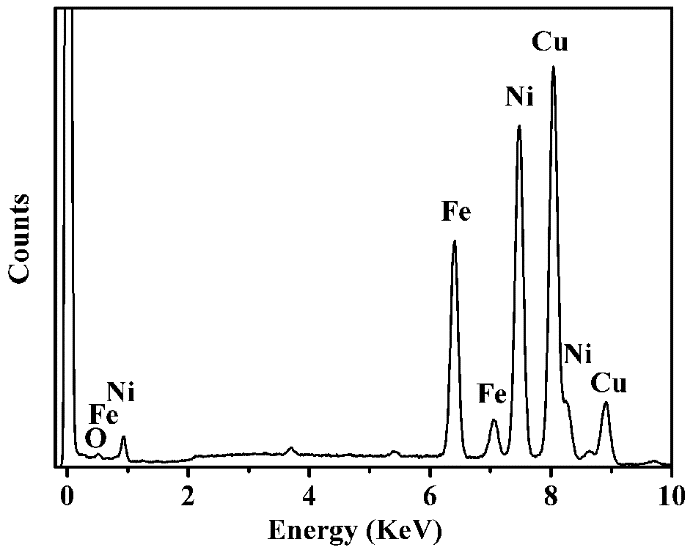


**Fig. S2.** EDS spectrum of the α-Fe_2_O_3_/NiO nanotubes.

**S1. Measurement of the conduction type of as-prepared NiO by** **thermal probe method**

The conduction type of as-prepared NiO was measured using thermal probe method. The schematic can be shown in Figure S3a. First, As-prepared NiO nanotubes powder was compressed to wafer (diameter: 1 cm, thickness: 0.5 mm). Then NiO wafer was placed on a cold Fe-Cr metal plate (room temperature, 20 °C). A electric soldering iron (ATTEN 936, SHENZHEN ATTEN ELECTRONICS Co., Ltd) which can continuously adjust the temperature was putted on the surface of NiO wafer. Subsequently, A multimeter (Fluke 233, Fluke Corporation) was connected between the iron head and the cold metal plate. We could decide the conduction type of as-prepared NiO according to the polarity of thermoelectromotive force. When the temperature difference exists between the NiO wafer and cold metal plate, the carrier in NiO wafer will diffuse from the hot side (NiO) to the cold side (metal plate). If the carrier is hole, the hot side will lack holes while the cold side will has excess holes. As a consequence, an electric field from the cold side to hot side will be formed. The potential of cold side is higher than that of hot side. Obviously, our testing result matched well with the situation of p-type semiconductor. As shown in Figure S3b, negative thermoelectromotive force (-75.2 mV~-141.7 mV) could be measured with the temperature of the hot side ranging from 200 °C to 450 °C. Therefore, we can evidence that NiO is p-type semiconductor with holes as the main species of charge carriers.


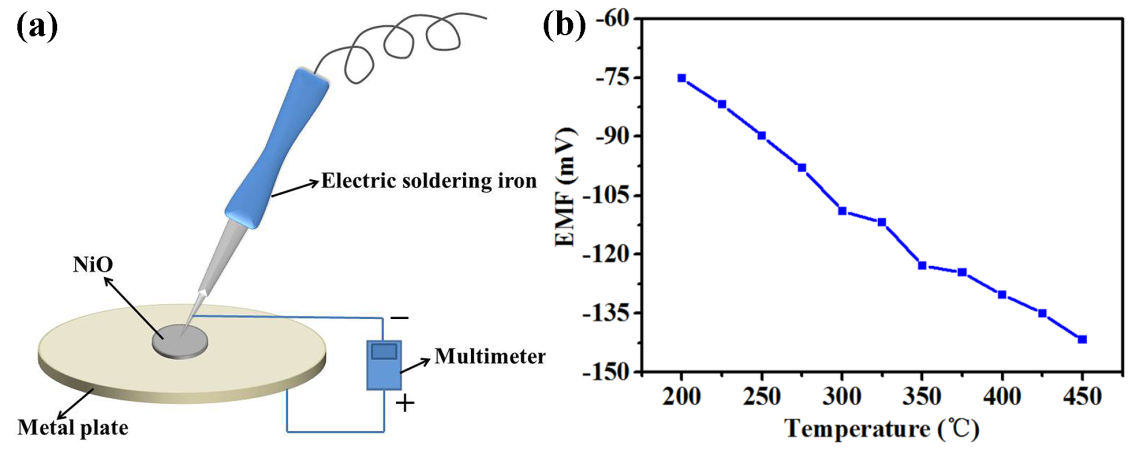


**Fig. S3.** (a) Schematic diagram of the thermal probe method; (b) Measured thermoelectromotive force with the temperature of the hot side ranging from 200 °C to 450 °C.


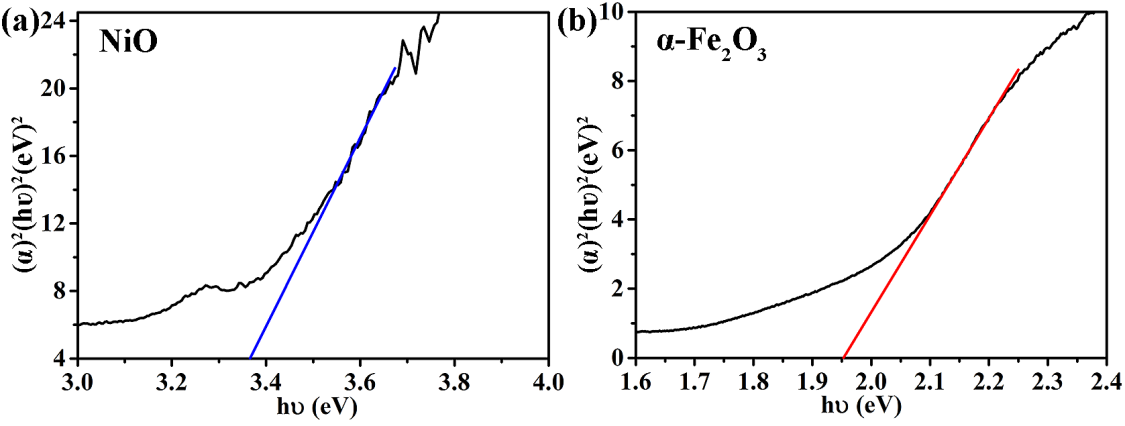


**Fig. S4.** Relationship between (α)^2^(hʋ)^2^ and hʋ for NiO (a) and α-Fe_2_O_3_ (b).


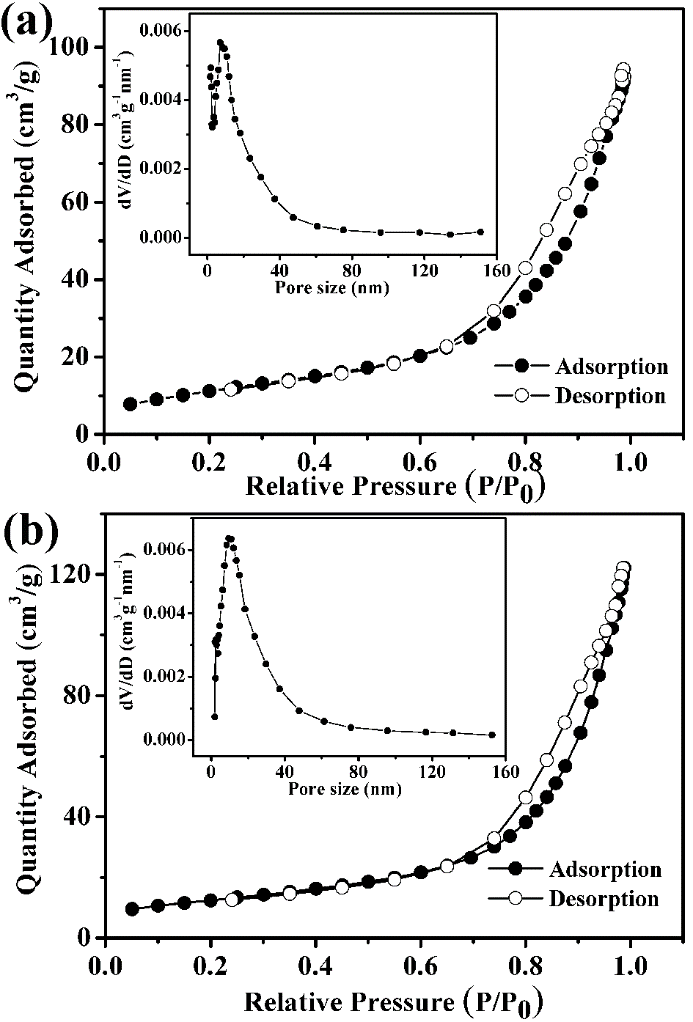


**Fig. S5.** N_2_ adsorption-desorption isotherms of (a) pure NiO and (b) α-Fe_2_O_3_/NiO nanotubes.

**
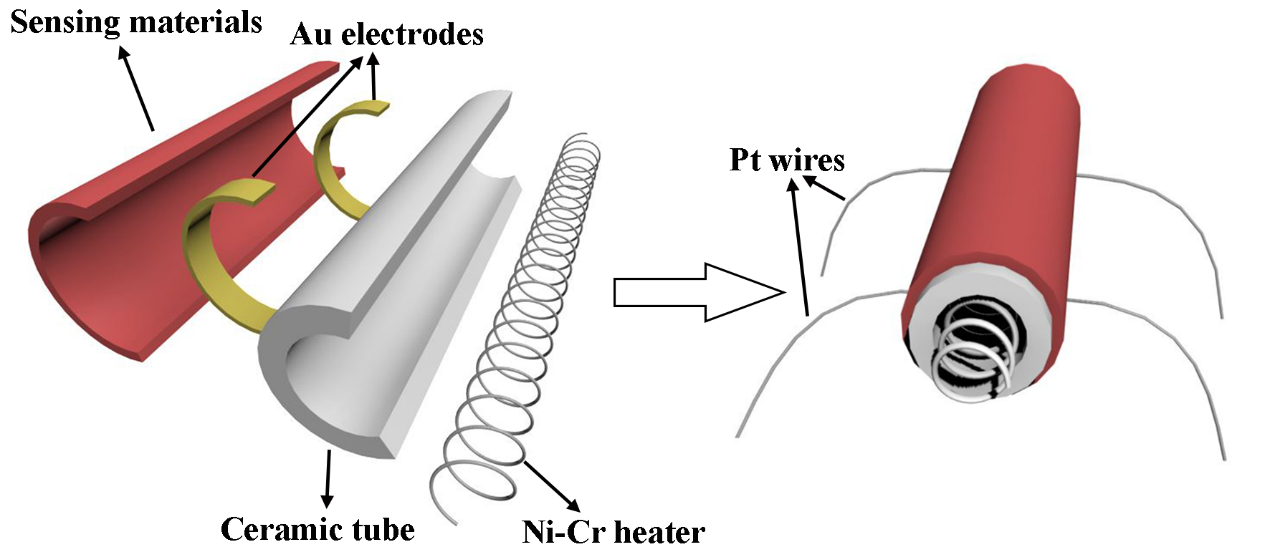
**

**Fig. S6.** Schematic diagram of the gas sensor.
